# Morphophysiological Responses of the Goat Mammary Gland to Water Scarcity in Arid and Semi-Arid Environments: Are They Enough to Generate Adaptation to New Climatic Challenges?

**DOI:** 10.3390/ani13243825

**Published:** 2023-12-12

**Authors:** Carolina Geldsetzer-Mendoza, José Luis Riveros

**Affiliations:** 1 Department of Animal Sciences, Faculty of Agronomy and Forestry, Pontificia Universidad Católica de Chile, Santiago 7820436, Chile; clgeldsetzer@uc.cl; 2 Escuela de Medicina Veterinaria, Facultad de Ciencias de la Naturaleza, Universidad San Sebastián Campus Bellavista, Santiago 7820436, Chile

**Keywords:** climate change, dairy goats, mammary gland, water intake, arid and semiarid territories

## Abstract

**Simple Summary:**

In the near future, several areas of the world will be affected by climate change, reducing their water availability. More than 90% of goats are found in Asia and Africa, and it is believed that these animals would be more resilient to climate change. Their milk is a good source of nutrients, contributing to the food security of the poorest and rural communities. Considering that milk is mostly water, it is essential to understand how the goat mammary gland can respond at a productive, cellular, hormonal, and blood level when faced with reduced water intake scenarios, as well as analyzing prospects in terms of more sustainable goat milk production.

**Abstract:**

Due to climate change, diverse territories of the planet will suffer from water restrictions. Goats are perceived as the most resilient ruminants in this scenario. So, various studies have focused on describing how a lower water intake influences milk production, especially in breeds adapted to desert environments. In water-stress situations, goats lose up to 32% of their body weight (BW), the rate of passage is reduced, and the digestibility of the feed increases. When goats consume water again, the rumen prevents hemolysis and osmotic shock from occurring. Regarding milk production, the response varies depending on the breed and the level of water restriction, maintaining the milk volume or reducing it by up to 41%. Systemically, it decreases the urinary volume and glomerular filtration rate, increasing blood osmolality and the vasopressin (ADH) concentration. Studies are scarce regarding changes in blood flow to the mammary gland, but there would be a reduction in blood flow velocity of up to 40% without changing blood pressure. New studies must be undertaken to determine which breeds or crosses are the best adapted to changing environmental conditions and to improve our understanding of the changes that occur at the morphophysiological level of the caprine mammary gland.

## 1. Introduction

Goats (*Capra hircus*) were among the first species to be domesticated, during the transition of the Neolithic period, 10,500–9500 years before the present, in the areas of southeastern Anatolia and the Zagros Mountains, now corresponding to Iraq and Iran [1,2,3]. They are widespread and primarily raised for the production of milk, meat, leather, and fiber [4]. It is estimated that there are 1.2 billion goats worldwide, of which 51.4% are concentrated in Asia and 43.4% in Africa [5]. Over the last two decades, the worldwide goat population has steadily increased by 44%, although the number of goats declined by 8% in Europe and 15% in China. Conversely, Africa’s population of goats has doubled (+89%), while the increase in Asia (+15%) and the Americas (+15%) has been more gradual [6].

Milk production has increased, reaching 20.7 million tons in 2021 [5], which is 75.9% more than in 1993, due to the increase in the goat population, which has reached nearly 219 million goats [7]. It should be noted that goats are still mainly used to produce meat [8], and goat milk represents only 1.3% of all milk marketed worldwide [9], with Europe being the leading market, where France, Greece, Spain, and the Netherlands stand out as significant consumers [8]. In addition, the increase in the world population and globalization have grown the demand for new foods [10].

India leads by producing 6 million tons of fresh goat milk annually, followed by Bangladesh, with 2.6 million tons [5]. However, due to goat rearing being associated with low-income countries in Africa, Asia, and the Americas, where the product is used for on-farm consumption and informal marketing of milk and milk products, the reported levels are likely underestimated. Goats are important because they can develop in unfavorable conditions and consume grass of low nutritional quality, providing an alternative for animal protein production and thus contributing to food security [11]. Furthermore, due to the effects of global climate change, including drought and erratic climate patterns, nowadays, nearly 20% of the global population lives in regions under serious risk from desertification [12]. Cattle are more complex to maintain, because they are less efficient biological converters and are less resistant to environmental stress, increasing small ruminants as a potential replacement. Due to their unique eating behaviors, goats could better utilize natural resources that are not accessible to other dairy species [7,13]. For instance, farmers in West Asia and North Africa opt for goats over cattle, due to their ability to adapt to arid climates and maintain higher levels of milk production [7].

Nutritionally, goat milk comprises 87.5% water and 13.2% total solids (4.5% fat, 3.6% protein, and 4.3% lactose), providing 67 kcal per 100 g of milk [14]. This composition offers multiple benefits to its consumers, which has improved its perception and has been confirmed by several studies [15,16,17], leading to increased demand. Among its characteristics, it is observed that it has a higher proportion of essential amino acids (52.5%) compared to dairy milk (46.7%) and sheep milk (48%), and compared with buffalo milk, goat milk has a higher concentration of most of the essential amino acids, except valine and isoleucine [18]. It has five times more folic acid than human milk and six times more than dairy milk [19]. It contains fat globules that are smaller than 3.5 μg, which facilitates its digestion. Furthermore, studies have reported that the presence of peptides inhibiting angiotensin-converting enzymes contribute to its antihypertensive effect. It also exhibits antimicrobial activity due to containing lactoferrin, lactoperoxidase, lysozymes, and peptide precursors to components of immunity [20,21]. Finally, it reduces the risk of anemia and bone demineralization because of its high levels of iron, calcium, and phosphorus [19].

Since milk is mostly water, it is important to consider this nutrient as essential. Therefore, it is recommended that goats have free access to drinking water. On average, 148 g water/kg BW^0.75^ is required [22]. However, it can also be calculated that they consume 10% of their live weight in water daily or 1.4–1.7 L of water per kg of feed consumed in dry matter (DM), or twice the amount consumed in DM. These values increase due to factors such as the ambient temperature, feed moisture, and milk production levels [22,23,24,25,26,27,28,29].

Due to the effects of climate change, access to water has become increasingly complex in certain regions. This trend is expected to worsen due to various factors including demographic growth and urban expansion, increased agricultural production, energy demand, and socio-economic development [12]. Therefore, previous studies have explained how water restriction affects milk production. The response in goats is influenced by breed, temperature, humidity, and duration–severity of restraint. Typically, an increase in osmolality leads to a decrease in milk production [30,31], as well as a decrease in fat and lactose content with a preservation of milk proteins [30,32]. At the blood level, an increase in the concentration of ADH is observed, which constricts the mammary artery, so a more significant reduction in milk production would be expected due to a decrease in blood supply, but, surprisingly, this is maintained in dehydrated goats [33]. Similar studies have been conducted in other species, such as camels, which were subjected to 3 or 6 days of water deprivation and continued to produce milk with a higher concentration of fat and protein without lactose variation. However, their productivity decreased (−15%) due to a reduced DM intake. Finally, it is noteworthy that in contrast to goats, camel BW loss is less (9% vs. 35%) [25,27]. A decrease in milk production of 11.6% and 16% has been observed in sheep and cattle that consume 60% of their daily water requirement [34,35].

Previous studies have explained the changes that the mammary gland undergoes during the different stages of lactation [36,37,38] and when goats are subjected to a restriction of water consumption [32,39,40,41,42,43]. This work aims to describe the progress made in understanding physiological responses of the goat mammary gland to new scenarios in the current environmental challenges of drought. Secondly, it aims to discuss physiological mechanisms, such as how the action of circulating and local IGF-1 is coordinated in the development of mammary ducts at different stages of lactation, to describe the influence of insulin on this growth factor, and to detail the mechanisms of osmoregulatory adaptation that the mammary gland has. Finally, some strategies will be proposed at different scales that will allow the sustainability of the goat production system and consequently strengthen the food security of marginal communities that are culturally closely linked to goat production.

## 2. Review Methodology

The literature search was conducted between December 2022 and June 2023 using PubMed and Google Scholar databases. The information search focused on studies related to the development and adaptations of the mammary gland and milk production in goats in relation to a reduction in water intake, considering the transport of milk elements, blood flow, and morphophysiological changes in goats and other ruminants. It summarized peer-reviewed papers written in English and Spanish. Publications covering breast cancer, mastitis, and lactation in non-ruminants were excluded due to the models used, which do not necessarily reflect goat mammary physiology.

Information on ruminant and caprine milk stocks and water stress levels is provided by the United Nations and FAO databases. The dairy goat production scenario was performed using a Google search. 

**Figure 1:**
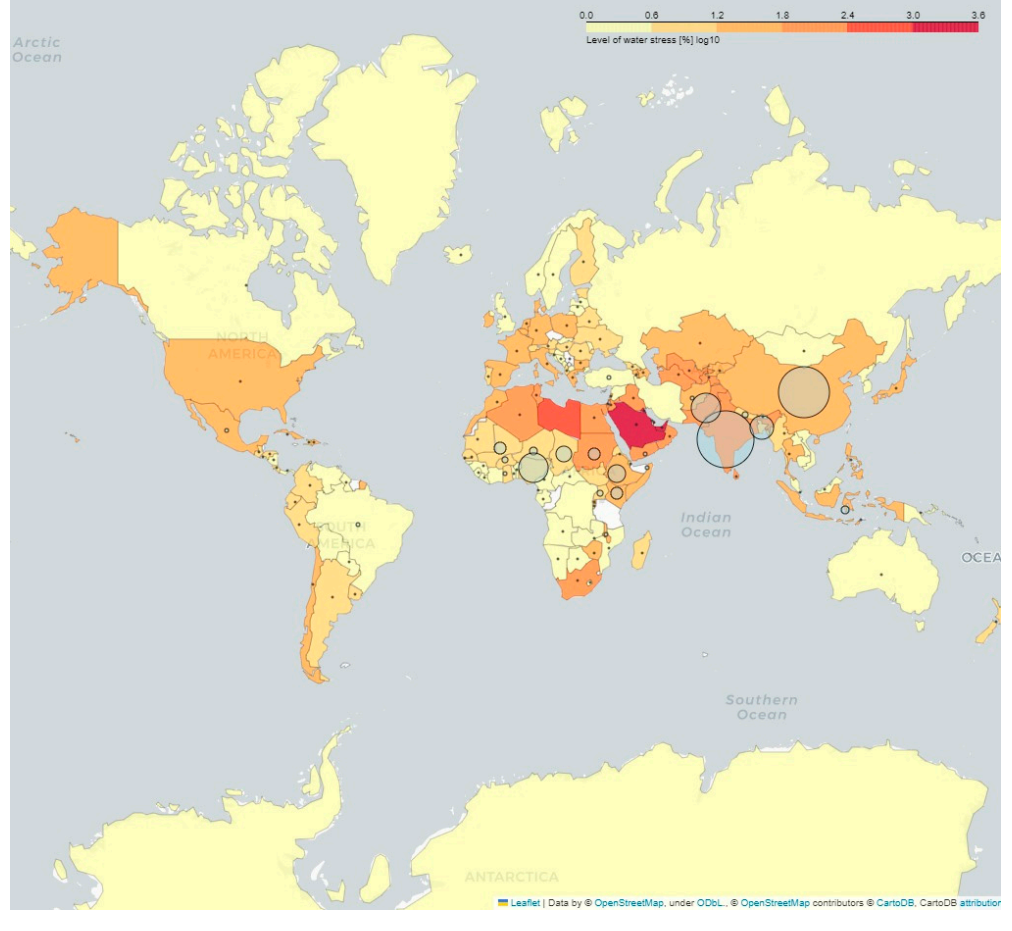
Worldwide goat distribution and level of water stress. Circles are the concentration of goats per country.

Figure 1 was created using the Folium library on Phyton 3.11.3.

## 3. Goat Dairy Production Scenario Worldwide 

Goats are crucial in underdeveloped countries because they are the main source of protein for children [44]. Asia is the leading producer of fresh goat milk worldwide, accounting for 58.7% of production, followed by Africa at 21.9%, Europe at 15.7%, and the Americas at only 4% [45]. India, China, Pakistan, Nigeria, and Bangladesh are the top five goat-producing countries (Figure 1) [5]. This contributes to the fact that two-thirds of these animals are found in tropical or subtropical areas of the world [44], with a significant part of these areas being hyper-arid, arid, and dry subhumid [46]. In certain instances, these regions also exhibit high water stress scores, consuming 25% or more of the available renewable freshwater resources. Globally, 33.8% of countries are in this situation, and in the case of India and China, for example, they have water stress scores corresponding to 66.49% and 43.22%, respectively [46].

The global description of dairy goat production systems is as follows: FAOSTAT [5] has recorded goat data for 179 countries, of which 113 have reported milk production. This is due to factors such as the focus on meat production for religious celebrations in some countries or goats not being included in agricultural censuses [47,48,49]. Milk production in various countries serves the purpose of self-consumption, nourishing underprivileged children, infant nutrition, and to a lesser degree, local retailing of whole milk, fermented milk, and cheese. Additionally, it is involved in agrotourism, with the exception of the Mediterranean region, where goat milk products are widely preferred.

Small-scale goat farmers typically operate in regions affected by drought (Figure 1), social inequality, and political conflict [50]. Owing to resources being limited, infrastructure is low-tech, and goats tend to be integrated with other species, such as sheep and cattle [13,51]. Regarding the information on the type of production systems by country, it is noted that 70% are characterized as extensive feeding systems, through grazing on private, communal, or state lands. Transhumance is a frequent practice in areas such as Algeria, Burkina Faso, Pakistan, Tunisia, and Turkey [52,53,54,55]. This system results in variable production rates and animal survival, as it hinges on regional rainfall patterns and agriculture [56]. The effective management of natural resources is crucial as overgrazing can lead to a reduction in the quantity and quality of milk produced, despite favorable agro-ecological conditions [57]. Consequently, some producers have shifted from grazing to a confinement system, a trend encouraged by government incentives [50,58]. A limited number of countries have a high proportion of intensive systems, and these systems often coexist with grassland systems. This can be observed in France, Spain, Italy [59], certain sectors of Greece, peri-urban areas of Liberia [60], and southern and southeastern Brazil [13].

The most used goats are Creole biotypes, and although their production is not comparable to specialized breeds, they are still used due to their adaptation to local conditions [55,56,61]. Projects have been implemented to introduce specialized breeds such as Saanen, Toggenburg, and Alpina and to cross them with Creole breeds. However, they have failed or had specific scenarios of success because dairy breeds have higher nutritional and care requirements than Creole biotypes [62]. For instance, 90% of goats in Iran are found in arid regions and have historically been raised for their fiber. However, in recent years, the Nadoshani breed and hybrids with European dairy breeds have an increased milk production [50]. In India, attempts have been made to introduce Saanen goats, with satisfactory results only in producers with good feed resources [63]. A similar case was observed in Chile, where in desert areas, Saanen goats produced only 36% of what was expected for the breed, which could be explained by the fact that, despite meeting their nutritional needs, they suffered from heat stress [64]. Akraim et al., introduced Cyprus Damascus goats and Murciano from Granada in Libya, which were crossed with local breeds such as Mahali. Some authors indicate that milk production was more significant in Mahali goats when crossed, increasing milk production by up to 60% [65].

## 4. Physiological Changes of the Goat Mammary Gland

From embryonic development to pregnancy, the goat mammary gland undergoes several changes that allow it to perform lactation physiologically and morphologically. These changes are known as sequential events: mammogenesis (breast development), lactogenesis (cell differentiation), galactopoiesis (secretion of milk products), and finally, mammary involution when lactation ceases and cell remodeling occurs [66]. The main changes that happen in the caprine mammary gland at a morphological level are detailed below.

### 4.1. Remodeling of Mammary Gland Tissue

Mammary development is a crucial factor in milk production and the overall productivity of goats. The most significant glandular growth occurs between 2 and 7 months of age and at a BW of 15–35 kg [67]. Variations have been observed between a virgin female and another in her second third of gestation, with a 70% increase in mammary volume and, at the molecular level, a 9-fold increase in the concentration of DNA and a 10-fold increase in the concentration of RNA [68].

Morphologically, the mammary gland is an organ composed of alveolar and tubular cells, forming the ducts, alveoli, and myoepithelium. The proportion of these cells varies according to the physiological stage of the goat, with more secretory cells observed in lactation and a more significant proportion of myoepithelial cells during involution [69]. The ordinal number of births also has an influence, increasing the number of alveoli. In primiparous females, there is approximately 5% less in the proportion of alveoli in relation to interstitial tissue compared to multiparous females. Additionally, there is variation in the ratio of mammary epithelial cells (MECs) to alveolar lumen. At the time of delivery, primiparous females have a 1:1 ratio and multiparous 1.6:1 ratio but tend to reach the same proportion on day 60 of lactation (1.7:1), which would indicate more activity of cell differentiation in primiparous females, which could be related to having a greater expression of IGF-1 [70]. These levels are regulated by insulin and growth hormone concentrations [71]. Prepuberal goats feeding ad libitum increased the proportion of connective and fatty tissue, while those under a dietary restriction had a greater development of secretory tissue [65]. A similar outcome was obtained in prepubertal goats with a higher proportion of concentrate in the diet since the concentration of prolactin was increased up to three times, generating an increase in the development and maturation of the mammary parenchyma [72]. In prepubertal and pregnant goats, a difference of up to 56% has been observed between those that received diets with low and high energy concentrations [72]. 

Another factor is described by Li et al. [73], who evaluated the reduction in the number of milkings on the mammary morphology, milking half mammary once a day and another three times daily. The results revealed an increase in the number of cells and a decrease in the size of alveoli that were in regression. Cells that were more cuboidal and lost their alveolar tubule shape experienced glandular regression and cell apoptosis due to milk stasis, accompanied by increased DNA fragmentation. This would be similar to late lactation, in which milk production decreases as milkings become less frequent [73]. Consequently, involution would be associated with increased intramammary pressure, cessation of sucking stimuli, and the presence of lactation inhibitory factor, a milk protein of 10 to 30 kDa [74]. Another factor would be the modification of the permeability of the tight junctions, which, after 24 h without milking, would increase between 50 and 75% and reduce cell activity by 31% [75]. 

Previously, Chedly et al. [76] observed that administering a calcium chelator in the mammary cistern, Na^+^, K^+^, and milk albumin were increased, which would be related to a disruption of the tight junctions of the gland tissue. Along with this, there is a decrease in the expression of E-cadherins, avoiding the transfer of ions and small molecules through the paracellular pathway, in case an alteration occurs in the tight junctions an unstable milk production will be generated [37]. In addition, to produce an alteration in the synthetic pathway of the mammary cells, the count of somatic cells and apoptotic cells increases, presenting a greater transcription of the Bax protein [76].

### 4.2. Hormone Secretion and Hormone Receptor Expression

Goats are short-day seasonal animals. During the spring–summer season, they are in their lactation stage [77]. Milk production and secretion are regulated by a complex interplay of hormones, including estrogen, growth hormone (GH), melatonin, progesterone, and prolactin (PRL).

The ovarian hormones, estrogens, and progesterone participate during puberty and gestation in cell proliferation, morphogenesis, branching of mammary ducts, and formation of the alveoli [78]. They also modulate the formation and maintenance of tight junctions by regulating the expression of claudins and occludins [37] The importance of these hormones has been proven in ovariectomized goats before puberty, which presented rudimentary and undeveloped mammary parenchyma, poor epithelial tissue growth, and a low presence of adhesion molecules. Consequently, mammogenesis would be altered [79].

Melatonin regulates circadian rhythms, but it is also involved in milk production. Its inhibitory effect has been observed in several species, but in goats, milk production varies according to the lactation stage rather than day length, indicating a lower concentration during lactation thresholds [77]. In the aforementioned study, a negative correlation was observed in melatonin, GH, and PRL blood concentration, resulting in an inhibitory effect on the mammary gland [77]. 

One of the essential hormones during mammogenesis and lactogenesis is PRL, whose secretion is stimulated by a reflex generated by sucking [80]. Although the mechanism in galactopoiesis is not well described in goats, it has been observed that in bovine cell cultures, the synthesis of caseins and lipids increases, and in lactating goats, when applying bromocriptine, which is an inhibitor of PRL, a reduction in milk production of 13% was observed [81]. PRL and GH have an important role in cell survival. PRL reduces the activity of IGFBP-5, and GH stimulates IGF-1, thus limiting cell apoptosis and the progress of gland involution [82]. It also participates in the activation of the regulatory factor STAT5, which triggers the expression of genes involved in milk synthesis and secretion, and it is critical in cell survival [83]. Finally, it has also been described that PRL expresses genes that are essential in the secretion of serotonin [80].

Another molecule involved in gland function is serotonin, a neurotransmitter with various receptors on epithelial, myoepithelial, and vascular endothelial cells [80,84]. Serotonin participates in the stability of the tight junctions of alveolar cells, in the regulation of milk protein production, in the modification of local blood flow, in the increase in myoepithelial cell contractility during milk secretion, and in the increase in calcium bioavailability [84]. 

Finally, leptin is essential in developing mammary ducts and glandular alveoli in virgin goats. At the beginning of gestation, it is found in high concentrations in adipocytes, duct cells, and the basement membrane around them. Subsequently, its concentration is reduced during late gestation and lactation since the cells are already completely differentiated and the inhibitory effect of PRL. At last, its concentration is increased again during breast involution [85].

### 4.3. Mammary Gland Blood Flow

Blood flow in goats is 5.5–7.2 mL/100 g of gland mammary tissue/min [86] (p. 492), and the ratio of blood flow to milk production, based on Fick’s principle of plasma clearance, in both goats and cows, varies between 200 and 1000:1, with 500:1 being a generally accepted value [86] (p. 499). Linzell [86] observed that the blood flow in the mammary gland ranges from 15 to 66 mL/100 g of breast tissue/min and that the result can vary up to 50% between one day and the next. Under normal conditions, as the day progresses, the blood flow of the external pudendal artery increases, with the lowest values obtained during the morning after milking. Burvenich [87] also indicated that the day before and the first of estrous, the blood flow is reduced by up to 33% because the voluntary intake of water and food is reduced. Also, it has been observed that the vascular network of the mammary gland undergoes changes in the last weeks of gestation, such as the increase in vasodilators such as CO_2_, which facilitate the arrival of nutrients at the beginning of galactopoiesis. In this way, the gland blood flow remains stable throughout lactation averaging 23.4 ± 5.06 cm/s [88]. 

Milk production and blood flow would not be correlated with the number of capillaries or their density. The size of blood capillaries triples during late lactation (189 ± 19 mm^2^) compared to early lactation (65 ± 19 mm^2^) and it is reduced by 61% during drying. However, the blood flow is only reduced by 50% [88]. The ordinal number of births also influences the ratio of blood vessels to the ECM and interstitial tissue. In primiparous goats, it is 71%, while in multiparous goats, it is 41%. In addition, during late lactation, the vascular tissue is reduced by 59% in primiparous and 31% in multiparous. This suggests that the regression of the vascular endothelium is likely a result of or related to MEC involution, given the association of circulatory tissue with nutrient contribution and waste product removal [70].

**Figure 2:**
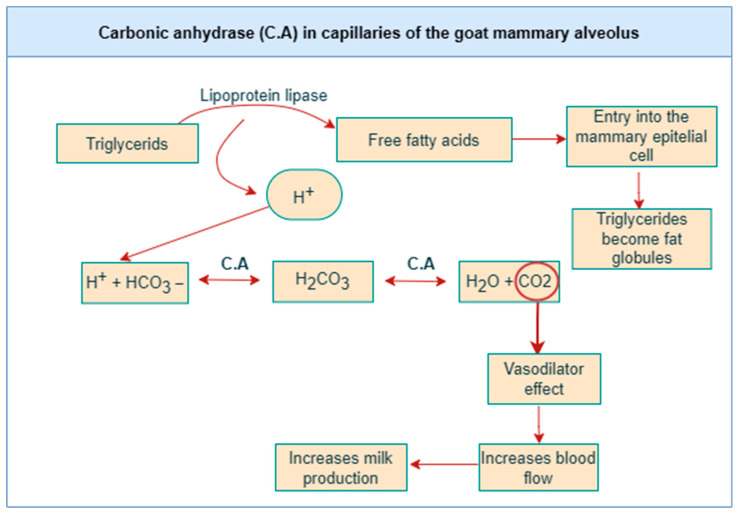
Participation of carbonic anhydrase (C.A) at the mammary gland level.

Based on the information provided by [38,88], it is proposed that carbonic anhydrase would have a fundamental role in the activity and blood flow of the mammary gland due to its ubiquity in blood capillaries. That would allow the functioning of lipoprotein lipase by reducing the acidification of blood. As a consequence of the activity of lipoprotein lipase, hydrogen ions are generated when triacylglycerol is hydrolyzed into fatty acids; carbonic anhydrase metabolizes H^+^, generating an increase in the CO_2_ concentration, which has a vasodilator effect. This would increase the local blood flow and consequently increases milk production given the greater flow of water and nutrients to the blood cells (Figure 2). In conclusion, it is described that there would be a coordinated adaptation between milk production, the mammary blood network, and the activity of carbonic anhydrase at the end of lactation, proponing that all these factors would decrease [88]. 

## 5. Physiological and Productive Changes in Goats under Water Intake Stress

### 5.1. Changes in Milk Production

It is described that goats are more efficient than cows in milk production under heat and water stress. They are more resilient [89], because it has been observed that at the same temperature humidity ratio (THR: 85), the reduction in production is 13% in goats and 33% in cows [90].

Several studies have considered factors such as breeds, periods, and water restriction levels, over responses to water intake reduction. For example, black and Somali Bedouin goats maintain their production against a water restriction for 24 and 48 h. Only on the fourth day, it decreases by 30% and 22%, respectively [27,43]. Damascus goats, also known as Shami, do not present changes in their BW or milk production because they keep their feed consumption stable [91]. On the contrary, in Swiss domestic goats, at 20 h of water deprivation, milk production is reduced by up to 41% [30], a response similar to that observed in Aardi goats [32].

In terms of productivity, research by [42] found that in Saanen and Alpina goats, after 16 h without access to water, they did not present a significant variation in volume and milk proteins, but in the percentage of fat and lactose [30,32]. Conversely, Damascus goats showed no alterations in milk composition under the same conditions [91]. Studies related to milk osmolality show different results; Mengistu et al. [31] observed an increase in their values, while Dahlborn [30] indicates that milk is isotonic to blood plasma under normal conditions. Dahlborn states that the decrease in milk production is caused by a lower water content in blood [30]. However, its results indicated a decrease of 45%, while osmolality only decreased by 8%. These suggest that there may be additional factors contributing to a decrease in nutrient absorption by the mammary gland.

### 5.2. Systemic Changes in Goats

By decreasing water consumption, forage intake is reduced by 18 to 25% [32,92], generating weight losses that vary between 10 and 25% of BW [30,32,40,43,93]. In lactating black Bedouin goats, after four days without access to water, their weight is reduced by 32%, 9% more than in non-lactating goats, which would be due to water losses through milk [94]. This would be an adaptive response of protection against an accumulation of food in the digestive system [95]. Although it is described that in goats adapted to arid environments, there is a reduction to 70% access to water, feed consumption remains stable because it would not be an extreme restriction. Therefore, the rate of passage of food, despite slowing down, favors an increase in the diet digestibility [96]. It should be noted that although the time in which rumen microorganisms perform fermentation increases and the synthesis of microbial protein and VFA increases, the rumen pH does not present alterations, remaining at 6.4; therefore, a water restriction would not be extreme. These factors would be related to a decrease in fecal excretion of DM, nitrogen, and neutral detergent fiber being important in maintaining the water economy [97,98]. This has also been described in camelids, whose metabolism is adapted to survive extreme situations of dehydration or water restriction, as they are able to spend weeks without consuming drinking water with access to sources of green fodder that provide them with this element through intake [25]. 

After restricting their water intake, goats can consume a high volume of it. The black Bedouin goat, for example, can consume approximately 40% of its BW in water without presenting pathophysiological effects since rumen slowly releases water to the rest of body, preventing hemolysis and osmotic shock [94,99]. Somali goats exposed to repeated restrictions have mechanisms for regulating their body fluids. After each rehydration process, the volume of water consumed is reduced by variations in blood ADH concentration [31].

One of the observed results from water deprivation is a decrease in the volume of urination and the glomerular filtration rate. Black Moroccan goats, lactating and non-lactating, normohydrated, produce 8 ± 2 mL/kg BW of urine with a glomerular filtration rate of 2.1 + 0.2 mL/min/kg. The latter is reduced by 44% in non-lactating goats after 48 h of deprivation with increased urine osmolality [93]. In blood, it is observed that there is an increase in creatinine and plasma urea [39,92], an increase in the osmolality of blood plasma due to an increased concentration of sodium and chloride [30,32,39,41,92,93,94], and an increase in blood ADH [39,40,93]. Blood renin is maintained at slightly low concentrations, which could theoretically prevent the vasoconstrictive action of angiotensin II in the gland and thus limit a reduction in milk production [94]. 

In the crossbreeding of Black Bedouin and Damascus goats, both breeds that have adapted to arid environments, by increasing water restriction progressively, there is a hemoconcentration, which increases the hematocrit from 33.7 to 47%, and ADH, which suggests better adaptation to water losses and greater water recovery. In addition, A-Tamimi et al. suggest that this is because the loop of Henle in the nephrons is longer than in other goat breeds, increasing the capacity of water resorption [100].

Physiological constants suffer modifications in goats subjected to water restriction and high temperatures [39]. An increase in the skin temperature of up to 0.40–0.97 °C has been observed without variation in rectal temperature, but the latter is significantly affected by the breed and the time and temperature of the day that the sample is taken [39,92]. Simultaneously, a decrease of up to 4.7–9.3% in the heart rate and an increase in the respiratory rate of up to 12.7–19.7% are observed [39].

### 5.3. Changes in Blood Flow on Mammary Gland

Few studies describe the effects of the blood flow level in mammary arteries under water intake restriction. Most information is associated with heat stress and food restriction. In the first situation, a reduction in the blood flow is observed due to an increase in norepinephrine and epinephrine, resulting in a decrease in the supply of nutrients to the gland and, therefore, a lower milk production and, finally, a lower input of oxytocin affecting its secretion [101]. In the second, a reduction in the cardiac output of 35% and an increase in peripheral resistance of 49% have been observed. As for the mammary gland, the blood flow after 48 h of food restriction is reduced by 69% (469 mL/min to 145 mL/min) and milk production by 72% [102]. 

Goats of the second and sixth lactation subjected to 48 h of water restriction presented a decrease in blood flow speed in the mammary vein of 20% on the first day and 40% on the second. Also, at the plasma level, there was an increase in ADH (1.3 ± 0.3 to 10.4 ± 1.7 pmol/L) [103]. No variations in blood pressure were observed in dehydrated goats, while feed deprivation reduced their heart rate and blood pressure [104].

Studies in other non-ruminant species observed rats subjected to a 50% food restriction. On day 14 of lactation, the cardiac output was reduced by 57%, from 136 ± 46 mL/min to 77 ± 2.9 mL/min. There was no variation in the lungs, kidneys, liver, and gastrointestinal tract. Milk production was reduced by 58%, from 72.8 ± 14.1 mL/d to 30.3 ± 30.9 mL/d [105]. Another study, also in rats under food restriction, observed that after 18 h without feeding, the blood flow of the left inguinal mammary artery was reduced by 48% (from 3.1 ± 0.2 to 1.5 ± 0.2 mL/min), and the glucose uptake was reduced by 88% (3.3 ± 0.4 μmol/min to 0.4 ± 0.1 μmol/min). In this study, no measurement of milk production was made [106].

## 6. Perspectives and Future Research Gap

In dairy goats under confinement, increasing the population mass, reducing the exchange rate, and increasing domestic food production would improve the system’s sustainability [107]. In addition, it has been observed that confined goats produce less methane than grazing ones and that the use of manure as fertilizer helps to sequester carbon [45]. 

Animal welfare experts are examining the adaptation of protocols and precision technologies from intensive and semi-intensive systems to extensive systems of small ruminants. The practicality of precision technologies, which offer greater insight into productivity, health, and well-being, has not yet reached the standards implemented in intensive systems [108].

Another factor would be related to increasing genetic diversity and selecting those individuals that are better adapted to changing environmental conditions and have better productive characteristics [45]. It has been suggested that in arid and semi-arid regions, Creole goats could be a viable option for local farmers, provided their feeding systems are enhanced. In this way, their milk production potential, which is generally unknown, would be reached. Consequently, it would work with animals adapted to the territories, taking advantage of its rusticity and ability to cover large grazing areas, being another alternative to crossing with specialized breeds [109]. In China, efforts are being made to conserve native breeds and create new hybrids with specialized breeds, such as the Guanzhong Dairy Goat, with a focus on modern and intensive systems [110]. Dairy goats have improved milk production in the arid regions of India and Albania by crossbreeding them with Saanen and Alpina goats [111,112]. It should be noted that studies generally only indicate nutritional characteristics of diet and milk production, but not variations in feed and water consumption. 

Goats are highly adapted to arid environments and rough conditions, producing milk despite nutritional adversities, making them an animal of great importance in food security, especially considering their role with small producers and poorer communities in underdeveloped countries. Despite this and the current knowledge about the consequences of climate change, resulting in higher temperatures and scarcity of drinking water resources, studies still need to be more conclusive in some mechanisms of physiological adaptations of these animals, considering those goats that live in areas where desertification processes are beginning.

An alternative in the future could be the selection of animals with a greater adaptation to arid environments and water scarcity, that present important traits such as their reproductive rate, maintenance of milk production, anatomical and morphological features, and tolerance to high temperatures [113]. However, conventional breeding strategies often prove ineffective in selecting these genes due to their low heritability (h^2^ ≤ 0.25), and that they are long-lasting, difficult, and expensive to accurately measure. Therefore, additional genome analysis and sequencing studies are necessary for small ruminants to identify regions of the genome linked to tolerance in desert environments [114,115].

It has been reported that under water stress, the calcium content in goat milk is reduced, which could be from to an alteration in the distribution of this mineral due to less blood flow and an alteration in the hormones involved in its metabolism [84,116]. It would be worthwhile to investigate whether a reduced water intake generates a lower availability of calcium associated with the tight junctions of secretory cells affecting the gland structure.

Studies suggest modifications in the mammary vascular network and the metabolic activity of epithelial cells during late lactation and involution. However, it is unclear whether changes in mammary metabolic activity govern changes in capillary morphophysiology or vice versa [70,88]. 

On a hormonal level, in ruminants under caloric stress during early and medium lactation, a decrease in the activity of dopaminergic neurons is described, which could be related to an increase in its antagonist, PRL, which would have a more thermoregulatory role than lactogenic from a decrease in milk production [117]. In the case of sheep under chronic stress is described as a side effect of the rise of dopamine generating a reduction in the concentration of PRL and reducing milk [118]. In the studies analyzed, there is no mention of the relationship between water stress or dehydration with the concentration of blood dopamine nor a relationship between the concentration of dopamine and PRL in blood. 

IGF-1 levels are influenced by the blood insulin concentration. However, studies measuring both metabolites in blood still need to be included, such as the participation of IGF-binding proteins (IGFBP) in the mammogenic action of IGF-1 in goats [71].

The studies analyzed were carried out under confinement conditions. However, the results could not be extrapolated in the pastoral systems, the most common form of goat production worldwide. This is because more variables influence animals’ physiological adaptations when subjected to a reduced water intake.

## 7. Conclusions

To conclude, despite the expansion of regions being negatively impacted by climate change, the population of goats and its milk production has significantly grown. Nonetheless, further investigation is necessary to describe hormonal changes and blood flow in goats’ mammary glands for a better understanding of whether these animals are morphologically and physiologically adapted to future water stress situations. Similarly, there is insufficient information to determine whether Creole goats or their hybrids with specialized breeds are the most water-efficient in dairy production in arid and semi-arid regions due to a lack of data concerning variations in water consumption and milk production in these crosses.

## Data Availability

Not applicable.
