# Correction: Geldsetzer-Mendoza, C.; Riveros, J.L. Morphophysiological Responses of the Goat Mammary Gland to Water Scarcity in Arid and Semi-Arid Environments: Are They Enough to Generate Adaptation to New Climatic Challenges? *Animals* 2023, *13*, 3825

**DOI:** 10.3390/ani14101445

**Published:** 2024-05-13

**Authors:** Carolina Geldsetzer-Mendoza, José Luis Riveros

**Affiliations:** 1 Department of Animal Sciences, Faculty of Agronomy and Forestry, Pontificia Universidad Católica de Chile, Santiago 7820436, Chile; 2 Escuela de Medicina Veterinaria, Facultad de Ciencias de la Naturaleza, Universidad San Sebastián Campus Bellavista, Santiago 7820436, Chile

Additional Affiliation(s)

In the original publication [1], there was an error regarding the affiliation for José Luis Riveros. In addition to affiliation 1, the updated affiliation should include: Escuela de Medicina Veterinaria, Facultad de Ciencias de la Naturaleza, Universidad San Sebastián Campus Bellavista, Santiago 7820436, Chile.

At the same time, the author needs to change the email address. The revised communication information is “Correspondence: joseluis.riveros@uss.cl”.

The authors state that the scientific conclusions are unaffected. This correction was approved by the Academic Editor. The original publication has also been updated.
